# Hæmorrhage after Lithotomy Stopped by Creosote

**Published:** 1842-03

**Authors:** 


*Hæmorrhage after Lithotomy stopped by Creosote.* By Dr. Daser.—In a case of lithotomy it was found impossible to arrest the hæmorrhage by any of the usual means, and no particular vessel could be discovered from which the blood might flow. The patient was at last reduced to the lowest ebb from the continued loss, and had already lost consciousness, when a sponge dipped in pure creosote was introduced into the wound, and pressed against the bleeding parts for an instant or two. The hæmorrhage was immediately arrested. No particular pain was experienced, no unpleasant symptoms followed: thin eschars were thrown off, and the patient recovered.—*Ibid.*, from *Journ. fur Chir. und Aug.*, 1840.

